# Transformative dimensions of resilience and brittleness during health systems’ collapse: a case study in Brazil using the Functional Resonance Analysis Method

**DOI:** 10.1186/s12913-023-09301-1

**Published:** 2023-04-10

**Authors:** Paulo Victor Rodrigues de Carvalho, Hugo Bellas, Jaqueline Viana, Paula de Castro Nunes, Rodrigo Arcuri, Valéria da Silva Fonseca, Ana Paula Morgado Carneiro, Alessandro Jatobá

**Affiliations:** 1 Instituto de Engenharia Nuclear (IEN), Rio de Janeiro, Brazil; 2 Centro de Estudos, Estratégicos Antônio Ivo de Carvalho (CEE), Fundação Oswaldo Cruz, Rio de Janeiro, Brazil; 3 Programa de Pós-Graduação Em Engenharia de Produção (TPP), Universidade Federal Fluminense (UFF), Niterói, Brazil; 4 Escola Nacional de Saúde Pública Sergio Arouca (ENSP), Fundação Oswaldo Cruz, Rio de Janeiro, Brazil

**Keywords:** Health systems resilience, Management, National health service, Infectious diseases, Public health emergencies

## Abstract

**Background:**

As health systems struggle to tackle the spread of Covid-19, resilience becomes an especially relevant attribute and research topic. More than strength or preparedness, to perform resiliently to emerging shocks, health systems must develop specific abilities that aim to increase their potential to adapt to extraordinary situations while maintaining their regular functioning.

Brazil has been one of the most affected countries during the pandemic. In January 2021, the Amazonas state's health system collapsed, especially in the city of Manaus, where acute Covid-19 patients died due to scarcity of medical supplies for respiratory therapy.

**Methods:**

This paper explores the case of the health system's collapse in Manaus to uncover the elements that prevented the system from performing resiliently to the pandemic, by carrying out a grounded-based systems analysis of the performance of health authorities in Brazil using the Functional Resonance Analysis Method. The major source of information for this study was the reports from the congressional investigation carried out to unveil the Brazilian response to the pandemic.

**Results:**

Poor cohesion between the different levels of government disrupted essential functions for managing the pandemic. Moreover, the political agenda interfered in the abilities of the system to monitor, respond, anticipate, and learn, essential aspects of resilient performance.

**Conclusions:**

Through a systems analysis approach, this study describes the implicit strategy of "living with Covid-19", and an in-depth view of the measures that hampered the resilience of the Brazilian health system to the spread of Covid-19.

## Background

In 2020 the world was hit by the first pandemic of the twenty-first century—the COVID-19 pandemic. With health systems worldwide pressured to their limits, many countries showed unpreparedness and incapability of tackling the rapid spread of the Sars-Cov-2 virus. Although unexpected events generate disturbances, health systems should become increasingly resilient to keep regular functioning during extraordinary situations like epidemics.

When functioning in normal situations, organizations adjust to complexity within a certain level of resilience, but information, resources and time are always finite, which affects such adjustments. Thus, when unexpected events occur—and healthcare is prone to unexpected events—the adjustments may not be enough to keep regular functioning while the system handles unwanted events [1].

The COVID-19 pandemic has revealed how constraints in policy-making and governance affect health systems resilience. In the Brazilian federalism, health services delivery is decentralized, as states are primarily accountable for covering their populations. This organization enables states and municipalities to organize their health networks according to their particularities, while the Federal Government is entitled to elaborate health policies and distribute the funding to implement the services locally.

Thus, different levels of government must collaborate cohesively to ensure that health services operate accordingly. In the case of the Covid-19 pandemic in Brazil, there never was such collaboration, and states and municipalities employed disjointed measures to tackle the pandemic.

Strategies for coping with Covid-19 in Brazil have been the subject of extensive debate [2, 3], and the positions, strategies and actions taken by the Brazilian Government diverged from most Brazilian States, international health authorities, and scientists.

The core of the Federal Government had never supported the adoption of known measures to tackle the spread of COVID-19. In the very first moments of the pandemic, the then sitting health minister was dismissed for diverging the President regarding subjects such as non-pharmaceutical measures, lockdowns, and dissemination of information. His substitute was also dismissed soon afterwards for similar reasons. It is important to note that the denialism of Federal authorities during COVID-19 was completely different from past positions, strategies and actions employed in the recent Yellow Fever, Zika, and Dengue outbreaks [4, 5].

The COVID-19 strategies and actions taken by the Federal Government’s officials—such as promoting agglomerations, anti-vaccines advocacy, discouraging mask use etc.—denotes that the federal government relied on the spread of the disease, sometimes referred to—distortedly—as ‘herd immunity’, although this strategy was never officially stated nor explicitly recommended.

Herd immunity is an essential concept in epidemiology, as with it only part of the population requires immunization. However, according to the literature, herd immunity still relies on a consistent strategy for vaccination rather than just promoting natural infection [6, 7]. Moreover, one must infer population immunity through systematic serological testing, another strategy that had not been consistently implemented in Brazil [8, 9]. In addition, unlike what has been claimed by some Brazilian authorities, herd immunity is hardly a measure to be taken in outbreaks like the COVID-19 pandemic. By the time this research was carried out, Brazil experienced approximately 700,000 deaths by Covid-19—more than 10% of world deaths, despite hosting only 2,7% of the world population [10].

One of the most expressive examples of the effects of denialism and its consequences to the resilience of the Brazilian Unified Health System (SUS) is the case of Manaus, in the Amazonas state, when patients hospitalized died due to lack of oxygen supply during the end of the second wave of COVID-19, in January 2021. Moreover, despite the health workers’ efforts to respond adequately to Covid-19, the SUS suffered with inadequate strategies, planning, and lack of resources and barely behaved resiliently, as various services struggled to maintain regular functioning during the pandemic [11].

Thus, this paper aims to uncover the details of the implicit strategy taken by the Brazilian Government to ‘live with COVID-19’, and we present an in-depth view of the case of Manaus, highlighting how such approach drove the measures that hampered the resilience of the SUS to the pandemic Furthermore, the present study provides evidence to understand how conflicting strategies in the different levels of government jeopardized the quality of care during the toughest moments of the Covid-19 pandemic. Based on lessons learned in this case, we provide recommendations for resilient strategies to cope with future crises.

## Methods

### Study design

This is a cross-sectional study based on the analysis of secondary data made public by the Congressional Commission that investigated the actions undertaken in Brazil to tackle the Covid-19 pandemic. In late April 2021, the Brazilian Senate started investigating the actions of the Federal Government during the Covid-19 pandemic. The hearings started on May 4th, and continued in the following months, until late October. Many authorities, politicians, businessmen were summoned and heard. The final report of the congressional commission was published on October 26th, 2021 [12].

The final report includes information from social media, newspapers, TV, official statements, as well as the contents of testimonies from different players—scientists, health authorities and workers, and the investigated persons. An extensive review of different documents, publications and other materials from the legal investigations was conducted by the commission and coded in the final report, making it the largest source of information concerning the actions taken by the Brazilian government to face the pandemic. Thus, this study takes the final report of the commission as its major source of information.

### Data analysis

The Functional Resonance Analysis Method (FRAM) [13] proved to be useful to represent the complexity of the situations explored in this study. FRAM models have been extensively used in representing the variability that affects the functions of complex sociotechnical systems [14–16]. Moreover, in this study the FRAM method was also used as a coding tool.

FRAM was originally developed to analyze technical systems, such as power plants, air traffic control, and production lines, but it has also been employed in healthcare [14, 17–19]. Although it was not specifically developed for social or political analysis, it can be used to model social systems by identifying their different components and interactions, providing in-depth descriptions of social norms, policies, and cultural practices that affect the behavior and responsivity to different events. For political science, FRAM could be used to analyze the interactions between different actors in a political system, such as parties, interest groups, and the media.

**Fig. 1:**
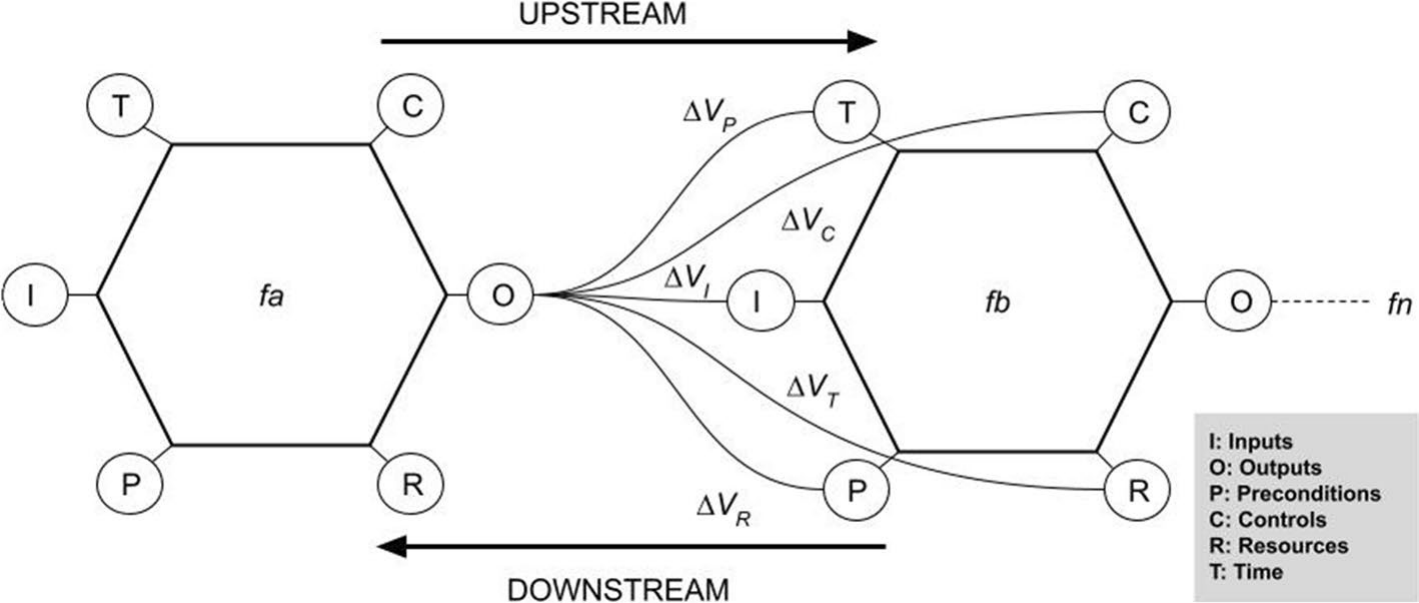
Variability's effects on FRAM upstream functions

FRAM organizes complex systems in their performed functions rather than their structures, while these functions are specified following the principles of joint cognitive systems. Specifications of FRAM functions are organized into six aspects as follows: inputs; outcomes; resources; pre-requirements; time; and controls (see the sample FRAM model in Fig. 1). Moreover, functions couple to each other, and such dependencies and interactions are non-linear, generating performance variability within the system.

Figure 1 shows two functions (*fa* and *fb*) coupling. The outputs of *fa* couple to different aspects of *fb*, i.e., the outputs of *fa* are used as aspects (pre-requirements, controls, inputs, resources, or time) by *fb,* generating variability expressed by Δ*V*.

Initially, we explored the recent literature to present a general FRAM model of the normal functioning of health systems while coping with Covid-19 in different countries (Slater, 2022). The key functions of the general model were organized according to the four abilities of resilient systems operationalized in the stress–strain model of resilience [20, 21]. Secondly, FRAM was employed to analyze the case of Manaus, and the functions proposed are based on the content analysis of the final report of the Congressional Commission that investigated the case.

## Results

### Resilient abilities as the cornerstones to live with COVID-19

**Fig. 2:**
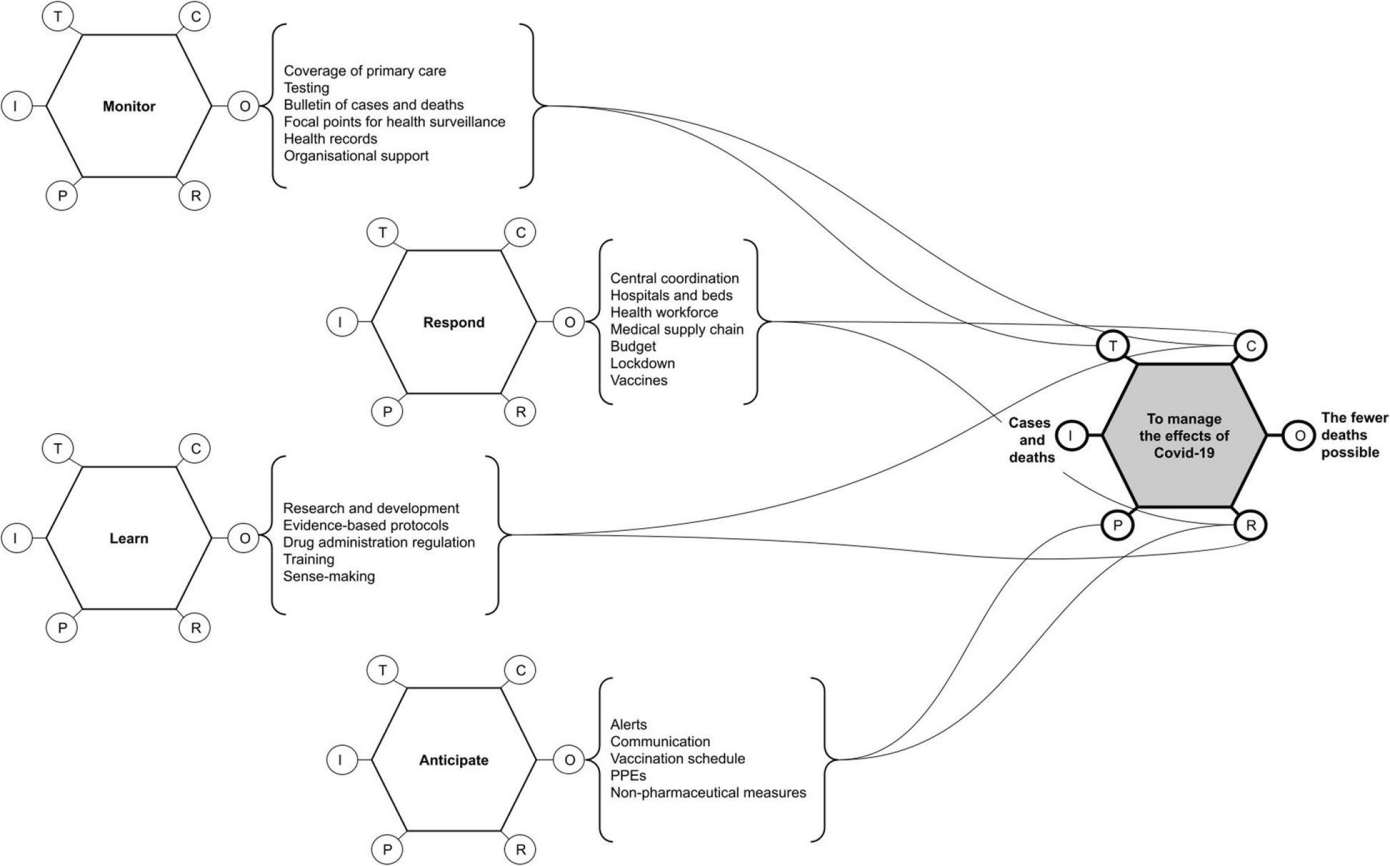
Attributes for "living with Covid-19" according to the resilience abilities

The FRAM model presented in Fig. 2 illustrates the overall aspects to cope with Covid-19 organized into the four resilience abilities proposed by Hollnagel [20]. Recent literature describes that as the pandemic spreads, health systems must operate to reduce the number of casualties to the minimum possible. Regarding unexpected events, old solutions proved to be useful. Where the unexpected happens, resilience involves the ability to continue functioning accordingly, and, if degraded, get back to its original condition. Systems are poised to adapt, thus resilience may become redundant as the organization develops new abilities in order to respond to unexpected events [22]. Therefore, the model in Fig. 2 is not supposed to exhaustively represent how health systems cope with events like Covid-19. Instead, it is a high-level overview of how the actions recommended by health authorities might lead to resilient performance.

Different aspects must be allocated to drive healthcare towards resilient performance to shocks like the Covid-19 pandemic. Monitoring and prompt response are essential and learning and anticipation must support long-term management of health services. One can see in the proposed model the importance of allocation of resources for increased testing capacity, and acute surveillance for outputting adequate monitoring [23, 24]. Similarly, adequate response outputs such as integrated supply-chain network, workforce, vaccines, hospitals, and acute coordination of resources control high-level management decisions. Moreover, eventual lockdowns were adopted successfully as a response measure in different magnitudes [3, 25, 26].

The ability to respond is based on how the system deploys, mobilizes, and generates responses to tackle dynamic demands. Resources must be allocated to stretch the capacity as situations cascade, and preconditions must be in place to enable satisfactory functioning.

The learning ability highlights the production and use of evidence-based protocols, as well as research and development to produce scientific evidence. Scientific evidence proved to be useful in communicating the non-pharmacological strategies and to mitigate vaccine hesitancy. Accepting non-pharmacological measures and avoiding vaccination hesitancy are influenced by trust in the authorities, safety and effectiveness of vaccines, trust in the health workforce and the public image of health systems. Furthermore, policymakers and government authorities who develop and communicate the strategies to ‘live with COVID’, such as vaccination plans, must be trustworthy. Moreover, Learning entails a knowledge base that enables an adequate medical supply chain, essential for behaving resiliently.

Resilience is dependent on the ability to anticipate potential bottlenecks or shortfalls. It is related to how systems exhaust the capacity to respond as challenges evolve. Common anticipation measures to tackle Covid-19 have involved social distancing and other non-pharmaceutical procedures, such as using masks and hands cleaning. They all rely on raising awareness and positive communication with the population, either through regular and social media, or through recurring alerts. Anticipation also relies on planned vaccination. Strategic plans are important to anticipate, strengthening the ability to cross boundaries and encouraging adaptive approaches in the face of adversity.

Learning from previous critical and/or adaptive experiences lead to reframing of models of adaptive capacity and reveals how extra adaptive capacity is necessary. Learning relies on awareness; thus, knowledge and sense-making occurs in a crucial learning process that defines what is known and what is not known. As learning progresses, knowledge is assimilated and integrated, raising awareness of the reality of the crisis.

### Running blind on a disaster scene: the scarcity of oxygen supply for Covid-19 patients in Manaus

In December 2020, a few months after the second wave of the pandemic, Manaus experienced a significant increase in the numbers of hospitalizations. Restrictive measures were adopted by the local government, but the residents rioted against such measures, causing the local government to withdraw the restrictive measures once employed.

Despite the official communication from the local government, the following increase in the number of Covid-19 cases wasn't overseen by the Ministry of Health, which only sent officials on January 3rd, 2021, when the situation in Manus was already catastrophic. Even so, the Ministry of Health's entourage, when confronted with the situation of increase of severe cases and scarcity of supplies, recommended "interventions for early treatment", according to the congressional commission report.

Such actions show the weakening of the monitoring and response abilities, as the warnings provided by the local focal point for health surveillance were ignored by the ministry's officials, and the lack of central coordination caused the scarcity of supplies for coping with the increase of severe cases. Measures to reduce contamination had just been abandoned, and the available hospital beds rapidly became insufficient, and the health system collapsed. The case is known as the "lack of oxygen", as hundreds of patients died due to the unavailability of oxygen supply for mechanical ventilation therapy.

**Fig. 3:**
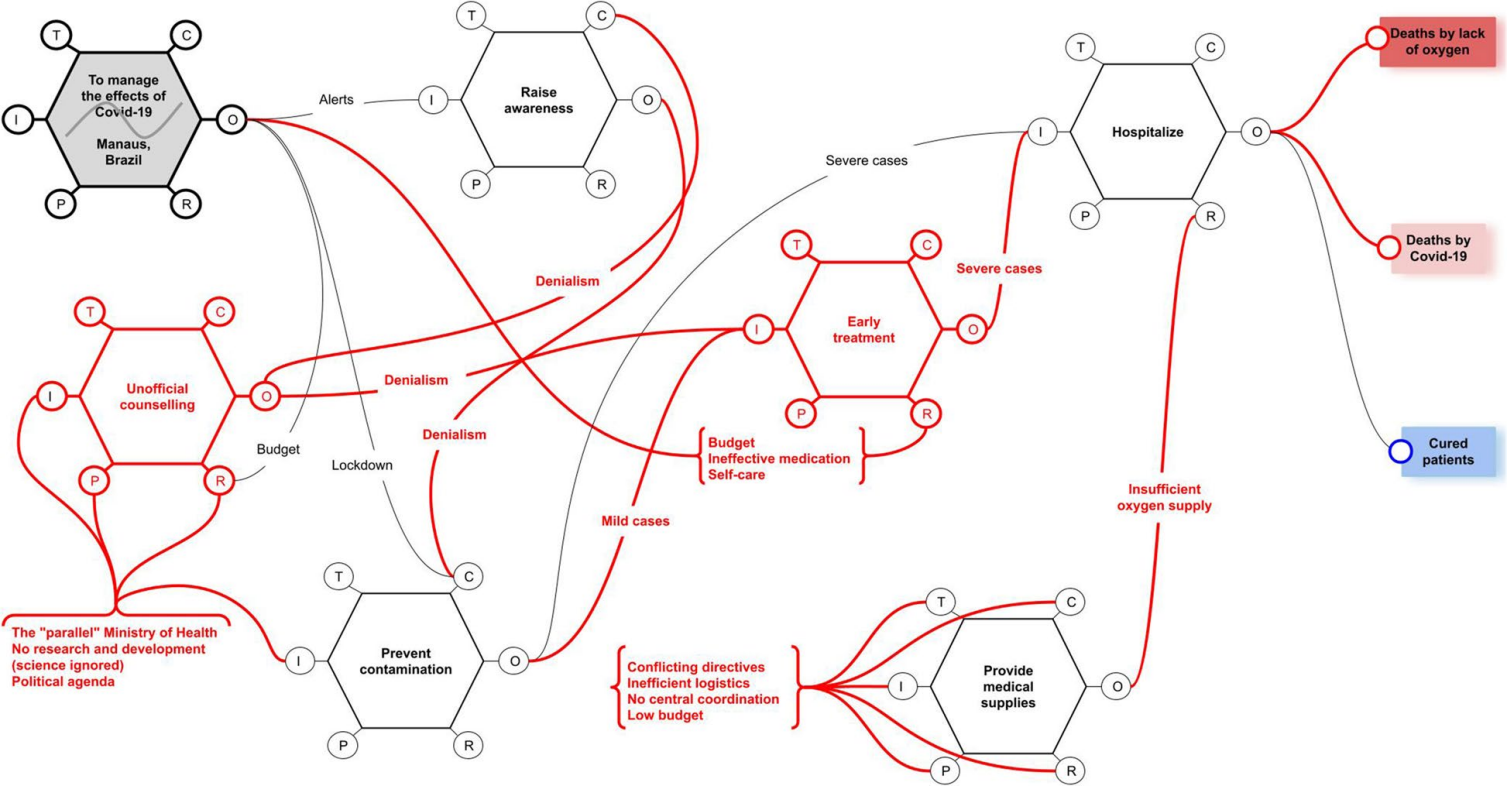
FRAM model of the collapse of the health systems in Manaus, Brazil

In red, the Fig. 3 illustrates the disruptions in the regular functioning of the system. According to the final report of the Congressional Investigation Commission, there are two functions that are not related to the general model (see Fig. 2), which were created by the Brazilian government: Early treatment; and Unofficial counseling. Those two functions generated variability on the aspects that hindered the normal functioning of essential features like raising awareness, providing medical supplies, and preventing contamination.

Fed by the so-called "Parallel Ministry of Health", the "Unofficial counseling" function disturbs the controls of the "Raise awareness" function by inputting denialist communications. Such denialism disturbs the general sense-making, and, consequently, hampers the measures to prevent contamination, as the population is confused by conflicting pieces of information. Unofficial counseling also disturbs the scientific consensus that is important to strengthen the learning ability, and puts response measures in jeopardy, like vaccines and lockdowns.

The report also relates the unofficial counseling to the adoption of measures referred to as "Early treatment", which consisted mostly in the use of ineffective medication, and self-care procedures—eventually supported by the mobile app "TrateCov". Early treatment was openly promoted by the Federal Government, and a significant budget was allocated to acquire medicines declared ineffective—or dangerous—by scientists and health authorities, like the WHO [27]. Thus, Covid-19 patients diverted effective oversight and underwent ineffective care, generating more severe cases—overloading hospitals—and deaths.

The lack of coordination also affected the supply of medical resources, especially the liquid oxygen for intensive therapy. As shown in the Fig. 3, variability disturbed the aspects of the "Provide medical supplies" function, as its inputs, controls, resources, preconditions and time were all affected by conflicting directives, poor logistics, and, consequently, outputting insufficient supplies to feed resources to the "Hospitalize" function, which caused an increase of deaths—in this case, not directly due to the infection, but due to the lack of oxygen for respiratory therapy.

## Discussion

The statements described in the investigation’s report indicate that the entourage sent to Manaus by the Ministry of Health sustained the strategy of spreading natural infection towards a "twisted" herd immunity. Moreover, the case of Manaus combined disastrous factors, like the unpopularity of non-pharmaceutical measures enforced by deliberate spread of denialism and misinformation regarding the outbreak, the insistence in promoting ineffective treatment, and the difficult logistics within a remote and hard-access region of the country.

Unwanted functions and their corresponding couplings (see in red in Fig. 1) were introduced as strategies in the regular systems’ operation, undermining the execution of essential functions. For example, we see that workforce and basic resources struggled to be mobilized due to the interference of unofficial counseling that introduced conflicting directives. Moreover, community engagement was undermined by misinformation, denialism, and fake news.

The European Commission lists resilient strategies employed within its Member-states to cope with the pandemic [28]. Several of the listed strategies corroborate the findings of Haldane et al. [29], since the success of these strategies depended on how health system were organized, governed and financed. In the next subsections we discuss our findings in the light of such strategies, indicating how Brazilian strategies lacked or were twisted during the collapse of the health system in Manaus in the Covid-19 pandemic.

### Coordination issues, governance, and influence of the political agenda

The SUS is organized according to the Brazilian federative pact that decentralizes governmental authority and, indeed, entail more responsive services [30]. However, central coordination of services may be more effective and efficient as central governments can manage resources to avoid overflows, correcting inequities.

 The lack of central coordination of efforts in Manaus makes it difficult to anticipate the effects of dubious approaches to face crises, as under conflicting goals variability remains uncontrolled. Although local health systems’ autonomy may improve performance by enhancing public oversight and accountability, the lack of central directives hampers the resilient performance as policy and decision making become more consuming.

According to the investigation report, a ‘Parallel’ Ministry of Health gave directions to the Federal Government regarding the measures to cope with the pandemic, which consisted essentially in early treatment procedures and herd immunity, considered ineffective, inaccurate, dangerous, and costly by the academia and most health authorities worldwide. Figure 2 depicts the ‘Live with Covid’ approach under which the Federal Government developed strategies to disseminate such concepts, which eventually conflicted with states’ directives, confusing the sense-making among the population and local governments [11, 31–33].

Primary care services are entitled to prevention and promotion of health processes, both of which are essential on the face of large outbreaks [34, 35]. As presented in the general model illustrated in Fig. 2, the coverage of primary care enables the monitoring function of the health system, as it entails dissemination of information, adequate awareness, mapping of risk factors, early detection and handling, and prompt referral.

The Manaus’ case has shown that known models were barely able to include the effects of political agenda in the engagement on the behavior of people regarding health actions. Without in-depth understanding on how health systems function, including issues regarding coordination, governance, and influence of political agenda, coping with crises becomes more complex.

### Monitoring, surveillance, and early warning

In the less affected countries, monitoring and acute surveillance showed to be essential for delivering timely and coherent response. As the pandemic exposed brittleness at both national and subnational levels, mobilizing monitoring systems, digital health detection, and expanding the capacity of early identification of cross-border threats is mandatory for resilient performance. Manaus is in the Amazonas state, at the Northern region of Brazil that has borders with underprivileged regions of Venezuela, Colombia, and Peru.

However, monitoring was blind in the case of Manaus, and response was delayed as the situation peaked rapidly. The provision of supplies was strongly constrained, overloading the hospitalization function of the SUS. Thus, herd immunity was never reached, and the costs of pursuing it, especially in lives, were very high.

### Delivery of timely response, effective political leadership, and maintaining routine services

According to Sagan et al. [28], the governance setup and the wider political context must be appropriately related to their non-health counterparts, mobilizing mechanisms that influence public policy. They must also steer how health systems are governed towards increasing responsiveness, resourcefulness, and capacity to learn in organizations and their leaderships. Moreover, as can be seen in the previous subsection, the ability to monitor affects the allocation of resources.

As the results of this study show, the leadership of local health authorities was undermined by the interference of the Federal Government, especially due its actions in communication and discouraging consensus on the strategies to be carried out.

Furthermore, the lack of oxygen supply impaired the functioning of other services, as patients in need of different therapies, either hospitalized or in home care, had their care discontinued. For example, 60 premature newborn children had to be transferred due to the imminent risk of oxygen supply shortage in neonatal intensive care units [36]. Moreover, the situation in Manaus disturbed the health system in other parts of the country, and supplies began to fall short as local health authorities—and even individuals—went after medical supplies without adequate monitoring from government authorities.

### Implementing vaccination programmes

Rather than the herd immunity advocated by the Federal Government – as detailed in the congressional commission’s report—effective vaccination is the actual way out of infectious diseases outbreaks, according to the public health expertise [9, 37, 38]. Since vaccines became available, successful experiences in tackling the pandemic were largely based on comprehensive vaccination strategies [28, 29, 39, 40].

As stated throughout the investigation, the purchase of vaccines was never a priority for the Federal Government. Negotiations for the acquisitions of vaccines started in different regions of Brazil in mid 2020, especially between the Butantan Institute, in São Paulo, and the Sinovac Company, manufacturer of the CoronaVac vaccine. The Ministry of Health delayed the negotiations, and rather than expanding its options to other manufacturers, it focused on the AstraZeneca vaccines, allegedly because of abusive prices, doubtful technologies, and complex handling of the other options.

Public investment usually played major roles in vaccine development and deployment, but investigators of the Congressional Commission considered that the Brazilian Ministry of Health neglected the acquisition of vaccines, which reinforces the prioritization of the herd immunity strategy from the beginning. Moreover, acute monitoring is a requirement for the provision of adequate supplies, logistics, and the mobilization of workforce for effective purchasing of vaccines. The most successful strategies employed national monitoring systems and real-time data, as well as coherent communication campaigns to engage the population an mitigate vaccine hesitancy [30, 41].

In addition to herd immunity not being scientifically recommended for tackling Covid-19 [37], essential procedures were barely adopted for an eventual strategy of living with Sars-Cov-2, such as the guarantee of sufficient medical supplies, strengthening of monitoring strategies, or the massification of non-pharmaceutical measures. The monitoring and responding abilities of the health system were strongly disturbed, and the actions performed went blind, either by interrupted flow of resources, or by misleading controls.

### Communication, anticipation, and transparency

Well-presented and broadly disseminated messages showed to be very useful to promote community engagement and compliance [28, 40, 42]. Using different channels also helped the most successful countries in increasing the reach of public health messages, while social media was largely used to access harder-to-reach audiences. However, diversity of channels entails higher possibilities for misinformation and confusion. Therefore, strategies to face fake news must be undertaken thoroughly, coordinating communication across multiple channels.

In Manaus, several warnings that opposed the strategy of the Federal Government, like the communications from local authorities and the WHO, were either ignored or contradicted, undermining the system's capacity to anticipate the disaster. With the complacency of Federal government, a wide fake news dissemination strategy through social media competed with developed scientific knowledge, resulting in confusing sense-making against evidence-based strategies to anticipate the virus’ spread.

It is important to highlight that the case of Manaus occurred one year after the pandemic was declared, when a third wave of COVID-19 was about to start in Brazil. Vaccines were still not available, but for the entire year of 2020 local authorities struggled to employ measures to prevent contamination, as the Federal Government acted constantly in opposition to social distancing, and in favor of natural infection.

January 2021 also marks the emergence of the first cases of the *Pi* variant in Brazil. All these events provided poor controls for monitoring and anticipation, combined with missing resources and insufficient conditions, especially for prompt response, making the health system unable to behave resiliently to the spread of COVID-19 in the region of Manaus in the case explored in this article.

### Towards a resilient future for the SUS

More than preparedness and strength, health systems must develop monitoring, response, anticipation, and learning abilities. In fact, strength and preparedness result from adequate monitoring, anticipation, and response, but developing a culture of resilience that enables continuous resilient performance relies strongly on learning from experience.

Learning lessons from past experiences is a key concept on proper responses to crises. The FRAM model in Fig. 3 shows that the activated functions have hardly produced outputs to foster continuous learning. In fact, research and development was undermined, and evidence-based protocols were dismissed, and sense-making was confused. Confusion made it difficult to take advantage of the ongoing experience to foster resilience. Moreover, the learning ability of the health system receives either implicit or explicit inputs, but in the case of Manaus, even implicit inputs were hindered by potential variability in the couplings, as the red colored symbols show in Fig. 3.

The SUS was conceived as a universal and equitable system, but also ‘integral’, i.e., health entails all levels of care and considers the people and their social, family, and cultural context. Despite historical and recurrent budgetary constraints and constant imbalance between demands and capacity, the SUS overcame recent political and economic crises, keeping stable coverage, especially to the poorest. Thus, the core principles of the SUS converge into resilience [32].

However further, appropriate, and well-managed investments in the SUS are needed at once, prioritizing neglected and deprived regions. Surveillance and monitoring systems must be improved, and primary care responsiveness must be enhanced, since it is a cost-effective level of assistance [43, 44], and prone to influence the social determinants of health. The Amazon region encompasses several remote, fluvial, and indigenous communities, and taking health services to these populations is challenging, thus, primary care plays a crucial role in providing outreach to underprivileged communities.

The experience of Manus in the Covid-19 pandemic also highlights the need for investments in governance and leadership to promote resilient performance in both extreme and regular situations. Health systems governance must consider the variable context and the usual political instability in Brazil, and strengthen trust between health systems, communities, and local authorities. However, such actions depend on the political will to prioritize and commit to health systems resilience as an essential attribute to ensure universal access and coverage towards comprehensive and long-term wellbeing – as listed in the Sustainable Development Goals [45] –, not just on the face of future threats and disasters.

Ensuring adequate allocation to funding, promoting the multi-professional nature of the health workforce, innovation, strengthening and expanding the coverage of primary care, and increased provision of complex services are essential aspects that will lead to the resilient performance of the SUS. In addition, it is necessary to restore the cohesion between different levels of governance on the implementation of public health policy.

## Conclusions and further work

This paper explores the actions performed by Brazilian authorities to manage the Covid-19 pandemic from a systems perspective, through a situated analysis of the collapse of the health system in Manaus, Brazil, in which medical supplies for basic Covid-19 therapy—like oxygen—went missing. A major finding of this study is that conflicting strategies were carried out, opposing federal and local governments, and disturbing the couplings between the essential functions of the systems for more than it could stretch, making it brittle.

Essential couplings were bypassed, while others were intercepted by irregular functions activated to take the inner strategy of herd immunity forward. Such functions worked with increased potential variability, and their couplings overloaded regular functions, especially the "Hospitalize" function, as the severe cases were potentialized. In other cases, potential variability hindered the inputs of functions aimed at the system's ability to respond to the pandemic accordingly—for example, shorting resources for providing medical supplies in favor of early (ineffective) treatment.

The findings are limited to the scope of the information available in the investigation conducted by the Congressional Commission. Thus, further investigation might be carried out to unveil additional details, especially concerning the economic and political implications of the decisions made both regionally and centrally, since the lack of central coordination was a major constraint for the health system's resilience.

The study is, therefore, limited to the line of events that mark the pandemic, although previous events, especially public policy employed as Brazilian President Bolsonaro's government plan, have affected the resilience of the SUS. The decline of the "More Doctors" program is just one example of public policy effects on the system's resilience prior to the pandemic, but further investigation will certainly provide more evidence on how the pandemic reveals historic brittleness in the Brazilian Unified Health System.

Regarding methodology, a systems analysis approach, such as the FRAM employed in this study, showed to be useful for unveiling the determinants of resilience and brittleness of the SUS in the face of a pandemic, providing important insights for transformative strategies to enhance the capacity of universal health systems to handle severe and chronic unexpected events. Moreover, FRAM made it possible to highlight the influence of political agenda in undermining the articulation of essential functions and in diminishing the cohesion between different levels of government, and how such scenario contributed to make Brazil the second epicenter of the pandemic.

Our analysis has shown that, despite the structures and functions available in a public health system, resilience depends on how such functions and structures operate. Resilient strategies to operate health systems functions are required to deal with major epidemics such the Covid-19 pandemic. The systems analysis presented in this article is focused on the case of Manaus, but many surrounding events throughout the pandemic have influenced the collapse of the SUS in different moments.

## Data Availability

All data generated or analyzed during this study are included in this published version.
